# Ecology of the Tick-Borne Phlebovirus Causing Severe Fever with Thrombocytopenia Syndrome in an Endemic Area of China

**DOI:** 10.1371/journal.pntd.0004574

**Published:** 2016-04-01

**Authors:** Zhifeng Li, Changjun Bao, Jianli Hu, Wendong Liu, Xiaochen Wang, Lei Zhang, Zhengmin Ji, Zhi Feng, Luxun Li, Aihua Shen, Xuejian Liu, Hongjun Zhao, Wenwen Tan, Jiangang Zhou, Xian Qi, Yefei Zhu, Fenyang Tang, Carol J. Cardona, Zheng Xing

**Affiliations:** 1 Nanjing University Medical School, Nanjing, China; 2 Jiangsu Provincial Center for Disease Prevention and Control, Nanjing, China; 3 Zhejiang Provincial Center for Disease Prevention and Control, Hangzhou, China; 4 Jiangning Center for Disease Prevention and Control, Jiangning, China; 5 Lishui Center for Disease Prevention and Control, Lishui, China; 6 Xuyi Center for Disease Prevention and Control, Xuyi, China; 7 Yixing Center for Disease Prevention and Control, Yixing, China; 8 College of Veterinary Medicine, University of Minnesota at Twin Cities, Saint Paul, Minnesota, United States of America; UC Davis School of Veterinary Medicine, UNITED STATES

## Abstract

**Background:**

Severe fever with thrombocytopenia syndrome (SFTS) is caused by SFTS virus (SFTSV), a tick-borne phlebovirus in family *Bunyaviridae*. Studies have found that humans, domestic and wildlife animals can be infected by SFTSV. However, the viral ecology, circulation, and transmission remain largely unknown.

**Methodology/Principal Findings:**

Sixty seven human SFTS cases were reported and confirmed by virus isolation or immunofluorescence assay between 2011 and 2014. In 2013–2014 we collected 9,984 ticks from either vegetation or small wild mammals in the endemic area in Jiangsu, China, and detected SFTSV-RNA by real-time RT-PCR in both questing and feeding *Haemaphysalis longicornis* and *H*. *flava*. Viral RNA was identified in larvae of *H*. *longicornis* prior to a first blood meal, which has never been confirmed previously in nature. SFTSV-RNA and antibodies were also detected by RT-PCR and ELISA, respectively, in wild mammals including *Erinaceus europaeus* and *Sorex araneus*. A live SFTSV was isolated from *Erinaceus europaeus* captured during the off tick-feeding season and with a high SFTSV antibody titer. Furthermore, SFTSV antibodies were detected in the migratory birds *Anser cygnoides* and *Streptopelia chinensis* using ELISA.

**Conclusions/Significance:**

The detection of SFTSV-RNA in non-engorged larvae indicated that vertical transmission of SFTSV in *H*. *longicornis* might occur in nature, which suggests that *H*. *longicornis* is a putative reservoir host of SFTSV. Small wild mammals such as *Erinaceus europaeus* and *Sorex araneus* could be infected by SFTSV and may serve as natural amplifying hosts. Our data unveiled that wild birds could be infected with SFTSV or carry SFTSV-infected ticks and thus might contribute to the long-distance spread of SFTSV via migratory flyways. These findings provide novel insights for understanding SFTSV ecology, reservoir hosts, and transmission in nature and will help develop new measures in preventing its rapid spread both regionally and globally.

## Author Summary

Severe fever with thrombocytopenia syndrome (SFTS) is an emerging hemorrhagic fever, caused by a tick-borne phlebovirus. Studies have found that a variety of domestic and wildlife animals can be infected by SFTS virus (SFTSV), but the natural reservoir host for the virus remains unclear. Although the SFTSV-RNA was identified in certain species of ticks or their larvae, contamination from their host animals cannot be excluded to be the source. We analyzed 9,984 ticks collected from vegetation or feeding mammals in 2013–2014 in Jiangsu province, an endemic area in China, and detected SFTSV-RNA in both parasitic and questing ticks. Interestingly, SFTSV-RNA was identified in larvae of *Haemaphysalis longicornis*, collected in vegetation without being blood fed, indicating a possibility of a vertical transmission of SFTSV in *H*. *longicornisis* in nature. We also detected SFTSV-RNA in four mammal species which may serve as natural amplifying hosts for SFTSV. In addition, we identified antibodies against the virus in two migratory bird species, suggesting wild birds, exposed to infected ticks, could spread the virus through flyways for long-distance transmission. These findings provide novel insights for understanding SFTSV ecology and transmission mechanism and help develop new measures to halt its rapid spread.

## Introduction

Severe fever with thrombocytopenia syndrome (SFTS) is an emerging infectious disease with a relatively high mortality, caused by the SFTS virus (SFTSV), a recently identified phlebovirus in the family *Bunyaviridae* [1]. The disease is characterized by high fever, a drastic reduction of platelets and leukocytes resulting in multi-organ failure in severe cases. The death rates reported have varied from 2.5 to 30%.

SFTSV was firstly isolated from a patient in Jiangsu province in the Eastern China in 2007 [2]. By the end of 2014, over 5,000 cases of human SFTS had been reported in 23 provinces in China [3]. There have been no data, however, to show the exact morbidity rate of SFTS in humans. Seroprevalence in humans varied from 0.44 to 7.2% based on reports in different epidemic areas [4,5]. The disease was also reported from Japan and Korea, where SFTSV strains were isolated, and a closely related virus called Heartland virus was isolated from patients with similar symptoms in the United States, which could be transmitted by ticks [6,7,8].

SFTSV is thought to be a tick-borne zoonotic virus [1,9,10], and has been detected in or isolated from several species of ticks including *Haemaphysalis longicornis*, *Amblyomma testudinarium*, and *Ixodes nipponensis* in China and Korea [11,12,13,14]. Heartland virus has also been isolated from *A*. *americanum* [15]. Previous studies conducted in Jiangsu and Shandong provinces of China showed that many domestic animals including goats, dogs, cattle, pigs, and chickens can be infected by SFTSV with no or only inconspicuous symptoms [13,16]. SFTSV-RNA has also been detected in larvae of *H*. *longicornis*, but contamination of the virus from the mammal hosts cannot be excluded since the larvae were collected from host animals [11]. A recent study showed that viral RNA could be detected in unfed larvae of *H*. *longicornis*, hatched from the eggs laid by infected adult ticks, demonstrating that SFTSV could be vertically transmitted experimentally. However, viral RNA was not detected in larvae collected in nature [17]. In addition, no live virus has been isolated from larvae or eggs [17]. Therefore, the natural reservoir, animal hosts and the mode of a vertical transmission for SFTSV have yet to be completely determined.

In this study, we conducted a comprehensive survey to elucidate SFTSV ecology in ticks and small wild animals in the SFTS endemic area in Jiangsu province. We detected SFTSV-RNA in both feeding and questing *H*. *longicornis* and *H*. *flava*. Interestingly, viral RNA was identified in larvae of *H*. *longicornis*, collected directly from the environment prior to a first blood meal, indicating that SFTSV could be vertically transmitted in nature. We also detected SFTSV-RNA in small wild mammals including *Erinaceus europaeus* and *Sorex araneus*, and detected viral antibodies in two migratory bird species, *Anser cygnoides* and *Streptopelia chinensis*. Infection of SFTSV in the wild bird species suggests that SFTSV could be transmitted long distances by them, which is also in agreement with a phylogenetic analysis reported earlier [18]. If further confirmed, it could explain why phylogenetically closely related viral strains have been identified in various regions geographically far away from each other.

## Methods

### 1. Study Design

#### 1.1 Case definition

A clinical case-patient was defined as a person who had fever, leukopenia, or thrombocytopenia. Other diseases, including hemorrhagic fever with renal syndrome, human granulocytic anaplasmosis and dengue fever were ruled out with serological assays. Paired serum samples were collected from all the survived clinical SFTS patients.

A laboratory-diagnosed case needed to meet either of the criteria: 1) virus isolation; 2) viral antibody seroconversion or a four-fold increase in antibody titers by indirect immunofluorescence assay (IFA).

#### 1.2 Ticks and small animal collection

**Fig 1:**
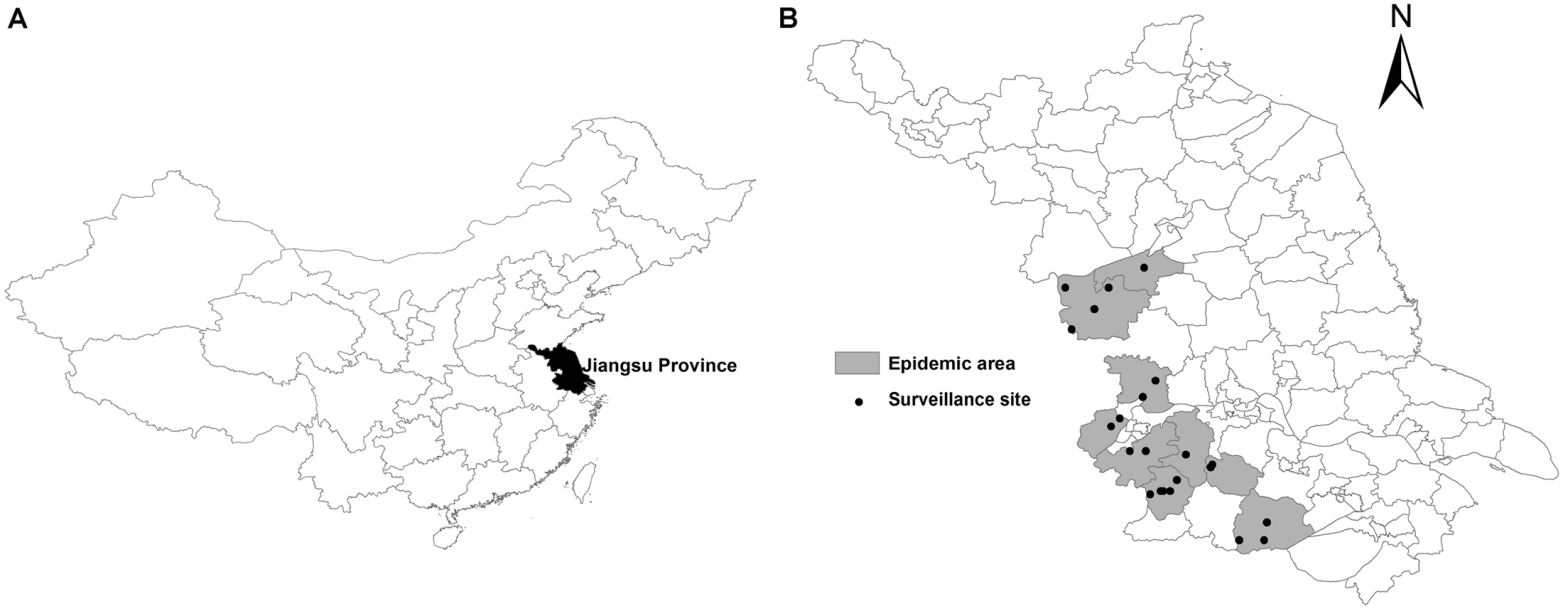
Distribution of Sampling Sites for Ticks and Wild Animals in Jiangsu, an SFTS Endemic Area. A, Map of P. R. China; B, Map of Jiangsu Province. Sampling sites were where SFTS cases were reported.

The study area was chosen in the western region of Jiangsu, the main SFTS epidemic area where human SFTS cases had been reported in a county in the past three years (Fig 1). Most of the area consists of a plain or low hill landform covered by grassland with small patches of broad-leaved forest, and possessing a temperate- subtropical climate. Collection of questing ticks was performed from March to December of 2013 and 2014 in 24 localities where SFTS cases occurred (Fig 1). Small wild mammals, birds, and feeding ticks were collected throughout the year in 2013 and 2014 in the same areas. For the feeding ticks, we collected them as tick-feeding season from March through October, and as off-tick-feeding season from November through next February.

All the sampling points (Fig 1B) were chosen near the areas where onset of human SFTS patients occurred within the past two weeks. Questing ticks were collected by using tick drags (1 x 1 m^2^ white cotton flannel cloth) through various habitats including deciduous forest and meadow. Tick collection was performed for 4 days during the first week of each month between 9:00 and 11:00 am and between 3:00 and 5:00 pm. All transects (approximately 200) were aggregated into 24 points (Fig 1B) by geographical positioning. Counts were standardized as the total number of adult ticks per flag-kilometer (flag-km). Data on tick abundance in each point was a total of the several transects examined in this area for a short period (4 days). The density and seasonal fluctuation were analyzed after classification of the specimens. Feeding ticks were collected from small wild animals. Ticks were pooled according to species and developmental stages (engorged or non-engorged larvae, nymphs, female and male adults) identified morphologically; each pool containing various numbers of ticks (1–30 for larvae, 1–20 for nymphs, and 1–10 for adult ticks/pool) was frozen and stored in liquid nitrogen. Small mammals were collected with live traps according to standard protocols as previously described [19]. Trapping grids were set up at sites adjacent to case households and in locations chosen to provide geographic diversity. Wild birds were obtained from local hunters.

All small animals were anesthetized using ketamine with a dose of 100 mg per kilogram weight by following the established safety guidelines for rodent captures and processing according to the Center for Disease Control and Prevention (Atlanta, GA) protocols [20]; blood samples were drawn from the retro-orbital sinus, and animals were sacrificed via cervical dislocation [21]. We necropsied the small animals on-site and collected hearts, kidneys, spleens, livers, lungs, and brains, which were subsequently stored in liquid nitrogen. Tick pools and animal organs were homogenized in 500 μl of TLR buffer supplied in the RNeasy kit (Qiagen, Germany) with a tissue lyser, Tissuelyser LT (Qiagen). The homogenates were used for RNA extraction and SFTSV-RNA detection.

### 2. Virological Methods

#### 2.1 SFTSV-RNA extraction and real-time RT-PCR

Total RNA prepared from the serum samples of the captured animals and the homogenates of the ticks or animal tissues were extracted using an RNeasy kit (Qiagen) according to the manufacturer’s instructions. The RNA was quantified with Nanodrop 2000 (Thermo Scientific, USA), aliquoted and stored at -70°C until use.

Real-time RT-PCR was performed using the QuantiTech RT-PCR kit (Qiagen). The primers were designed as previously described and used in a one-step real-time RT-PCR [19]. The forward (S-for)/reverse (S-rev) primers and MGB probe (S-pro) used in the real-time RT-PCR were targeted to the S segment of the viral genome [19]. Conditions for the reaction were as follows: 50°C for 30 min, 95°C for 15 min, 40 cycles at 95°C for 15 sec, and 60°C for 1 min. Amplification and detection were performed with an Applied Biosystems 7500 Real-time PCR system (Applied Biosystems, Foster City, CA). Data were analyzed using the software supplied by the manufacturer.

#### 2.2 Nested PCR detection

Ticks and animal tissues with positive results by real-time RT-PCR were further examined for SFTSV RNA S segments by nested RT-PCR, amplifying a 467-nt fragment from the small (S) segment of the viral genome. The first round of nested RT-PCR was performed using a One Step RT-PCR Kit (Qiagen, Germany) with designed outer primers, SF1: (5’-CCTTGACCCTGCTTTGAT-3’) and SR1: (5’-GCCACTTTACCCG AACAT-3’), which were designed using Primer Premier 5.0, under the following conditions: an initial step of 30 min at 50°C for reverse transcription and 15 min at 95°C for denaturation, followed by 35 cycles of 20 sec at 95°C, 40 sec at 50°C, and 30 sec at 72°C, and a final extension step of 5 min at 72°C. The second round of PCR was performed with primers which were used in a conventional RT-PCR in our previous study [19]. The reaction was performed in the same condition as the first round PCR. The products of the second round PCR were sent to Sangon Biotech Co., Ltd (Shanghai, China) for DNA sequencing with Sanger method using an automated ABI 3731XL DNA sequencer.

#### 2.3 Virus isolation

SFTSV-RNA positive serum samples from 67 humans and 7 animals and supernatants or homogenized tissues from viral RNA-positive animals or ticks were used to inoculate Vero and DH82 cells for virus isolation as previous described [1]. Briefly, the serum or supernatants of homogenized tissues (in Modified Eagle Medium, MEM) were inoculated onto the Vero cell for two weeks. The culture supernatants were harvested and re-inoculated onto fresh DH82 cells. Cytopathic effect (CPE) on inoculated DH82 cells was examined daily under a light microscope. Isolated viruses were further identified by real-time RT-PCR.

#### 2.4 SFTSV genome sequencing and phylogenetic analysis

The large (L) segments of SFTSV strains isolated from ticks and animals (S1 Table) were amplified by RT-PCR using the primers, based on published sequences of the SFTSV strains, described in the previous study [1]. The L gene encodes the viral RNA-dependent RNA polymerase and is considered more conserved than other segments in the genome. The products were sent to Sangon Biotech (Shanghai, China) for Sanger DNA sequencing. The termini of SFTSV-RNA segments were determined with the FirstChoice RLM-RACE Kit (Invitrogen, USA), and percentage similarities of nucleotide identity were calculated using Clustalx software. A phylogenetic tree for the L RNA segment was constructed with the neighbor joining method using the software MEGA 5.1.

### 3. Serological Methods

#### 3.1 ELISA for SFTSV antibody detection

Serum samples from animals were tested for SFTSV antibodies including IgG and IgM with a commercial double antigen sandwich ELISA kit from Xinlianxin Biotech (Wuxi, China) [22,23]. For initial screening, an undiluted serum sample was used to determine whether the sample was positive for viral antibodies. Positive serum samples were further diluted in a 2-fold serial dilution starting at 1:2 for the assay to obtain endpoint titers determined by the cutoff values set by positive and negative controls as provided with the ELISA kit.

#### 3.2 Immunofluorescence Assay (IFA)

SFTSV-specific IgG antibodies were detected in all human sera by IFA as previously described [24]. Twenty microliter of diluted (1:2 to 1:1280) serum samples was added to the cell-spotted coverslips with viral antigens and incubated for 45 min at 37°C. After washing, 20μL of FITC-conjugated goat anti-human IgG (Abcam, UK) diluted 1:80 by us with Phosphate Buffered Saline (PBS) containing Evans Blue (1: 20,000) was added and the samples further incubated for 30 min at 37°C. After three washes, the slides were mounted in glycerin and examined under an immunofluorescence microscope.

### 4. Ethics Approval

Small wild mammals and wild birds were captured and handled in strict accordance with good animal practice according to the Animal Ethics Procedures and Guidelines of the People’s Republic of China (Regulations for Administration of Affairs Concerning Experimental Animals, China, 1988). Handling and sampling including tick and blood collection were also approved by the Animal Ethics Committee, Jiangsu Provincial Center for Disease Control and Prevention, with the certificates No. SCXK[su] 2012–0021 and No. JSCDCLL[2012]039. The certificates also provided the permission on the protocol for sampling wild birds including *Anser cygnoides*. The hunters, who procured the wild birds for recreational purpose, agreed to provide the birds for sampling upon request. The hunters were not part of the research team but were aware of the nature of the study. Thirty-one blood samples were collected from the SFTS patients with written informed consent obtained from all participants. The study involved in human blood sample collection and virus isolation was pre-reviewed and approved by the Ethics Committee of the Jiangsu Provincial Center for Disease Control and Prevention (Certificate No. JSCDCLL[2011]038). However, sample collection from the SFTS patients was not specifically for this study; instead the collection and the Certificate were for an existing project aiming at isolating the virus from the human patients.

## Results

### SFTS Endemic Area and Sampling Sites

Sixty-seven SFTS cases were reported in the region of Jiangsu province (Fig 1A) between 2011 and 2014, which were confirmed by virus isolation or serological diagnosis, which included antibody seroconversion or a four-fold elevation of the IgG antibodies against SFTSV in paired serum samples by IFA. Among the patients, 9 of 67 (13.4%) had experienced tick bites before the onset of disease. We designated the region where SFTS cases were identified to be the SFTS endemic area and sought to conduct a comprehensive study of SFTSV ecology in the selected sampling sites (Fig 1B).

### Collection of Questing and Feeding Ticks in the SFTS Endemic Area

A total of 9,984 ticks (1,518 larvae, 5,336 nymphs, 1,412 male adults, and 1,718 female adults) were collected from 2013 to 2014 (3,240 in 2013 and 6,744 in 2014) from vegetation or wild mammals or birds in 24 localities (Fig 1; Tables 1 & S2). The tick samples were identified as belonging to four species: *Haemaphysalis longicornis*, *H*. *flava*, *H*. *concinna*, and *H*. *doenitzi*. Of the identified ticks, *H*. *longicornis* (85.3%, 8,520/9,984) was the most abundant species collected, followed by *H*. *flava* (9.6%, 958/9,984), *H*. *concinna* (3.7%, 366/9,984), and *H*. *doenitzi* (1.4%, 140/9,984).

**Table 1:** Detection of SFTSV RNA in Various Stages of *Haemaphysalis longicornis* and *Haemaphysalis flava* Ticks Collected from Vegetation and Animals in 2013–2014 in Jiangsu.

Sources	Species		2013				2014			
			Ticks	Pools	Positive pools	MIR (%)*	Ticks	Pools	Positive pools	MIR (%)
**Vegetation**	***Haemaphysalis longicornis***	**Larva**	250	10	0	0	530	30	2	0.4
		**Nymph**	910	92	2	0.2	1972	150	10	0.5
		**Male**	224	16	2	0.9	672	96	6	0.9
		**Female**	196	14	0	0	804	100	5	0.7
		**Subtotal**	1580	132	4	0.3	3978	376	24	0.6
	***Haemaphysalis flava***	**Larva**	14	2	0	0	70	4	0	0
		**Nymph**	70	6	1	1.4	212	16	1	0.5
		**Male**	44	6	0	0	38	4	0	0
		**Female**	32	4	0	0	64	8	0	0
		**Subtotal**	160	18	1	0.6	384	32	1	0.25
**Animals**	***Haemaphysalis longicornis***	**Larva**	284	12	2	0.7	226	10	2	0.9
		**Nymph**	618	48	2	0.3	1064	76	7	0.6
		**Male**	76	10	0	0	210	16	2	1.0
		**Female**	110	14	0	0	374	28	2	0.5
		**Subtotal**	1088	84	4	0.4	1874	130	13	0.7
	***Haemaphysalis flava***	**Larva**	46	4	0	0	26	1	0	0
		**Nymph**	94	10	1	1.1	126	8	1	0.8
		**Male**	4	1	0	0	50	6	0	0
		**Female**	6	1	0	0	62	8	0	0
		**Subtotal**	150	16	1	0.7	264	23	1	0.4
**Total**		**Larva**	594	28	2	0.3	852	45	4	0.5
		**Nymph**	1692	156	6	0.4	3374	250	19	0.6
		**Male**	348	33	2	0.6	970	122	7	0.7
		**Female**	344	33	0	0	1304	144	7	0.5
		**Total**	2978	250	10	0.3	6500	561	37	0.6

*MIR: Minimum infection rate, based on the formula, number of positive pools/total number of ticks tested.

Male: Male adult ticks; Female: Female adult ticks

Questing ticks started to appear in March, reached peak density in June and declined afterwards through September. A total of 6,360 questing ticks were collected (1,910 in 2013 and 4,450 in 2014), including 886 larvae, 3302 nymphs, 1,042 male adults, and 1,130 female adults (Tables 1 & S2).

### Collection of Ticks from Wild Mammals and Birds in Tick-Feeding and Off-Tick-Feeding Seasons

To collect feeding ticks and identify potential wild animal hosts for the virus in nature, we captured small wild mammals and birds during our survey in 2013 and 2014. During the two consecutive years, 1,283 small wild animals representing 6 species of rodents, 1 shrew, 2 wild birds, and 1 hedgehog were captured during the tick-feeding or off-tick-feeding season. The most frequently trapped animal species were *Mus musculus* (21.0%), *Sorex araneus* (14.3%), *Rattus flavipectus* (13.5%), *Rattus norvegicus* (11.5%), and *Erinaceus europaeus* (12.4%) (Table 2). A total of 3,624 ticks were collected from these mammals and birds, including 632 larvae, 2,034 nymphs, 370 male adults, and 588 female adults. In addition, blood samples were collected and sera were prepared from these animals for either SFTSV isolation, viral antibody or viral RNA detection.

**Table 2:** Detection of SFTSV RNA and Antibodies in Wild Mammals and Birds Captured in 2013–2014 in Jiangsu Province.

Animal Species	*Tick-feeding Season			#Off Tick-feeding Season			Total		
	Animal No.	^&^Viral RNA Pos. No. (%)	Viral Ab Pos. No.(%)	Animal No.	^&^Viral RNA Pos. No. (%)	Viral Ab Pos. No.(%)	Animal No. (%)	^&^Viral RNA Pos. No. (%)	Viral Ab Pos. No.(%)
*Mus musculus*	168	1(0.6)	3(1.8)	102	0(0)	1(1.0)	270(21.0)	1(0.4)	4(1.5)
*Rattus norvegicus*	82	0(0)	1(1.2)	66	0(0)	1(1.5)	148(11.5)	0(0)	2(1.4)
*Rattus flavipectus*	102	0(0	2(2.0)	71	0(0)	0(0)	173(13.5)	0(0)	2(1.2)
*Apodemus agrarius*	39	0(0)	1(2.6)	25	0(0)	0(0)	64(5.0)	0(0)	1(1.6)
*Niviventer coxingi*	36	0(0)	1(2.8)	28	0(0)	0(0)	64(5.0)	0(0)	1(1.6)
*Rattus fulvescens*	46	1(2.2)	3(6.5)	20	0(0)	0(0)	66(5.1)	1(1.5)	3(4.5)
*Sores araneus*	102	2(2.0)	5(4.9)	82	1(1.2)	2(2.4)	184(14.3)	3(1.6)	7(8.3)
*Erinaceus europaeus*	93	5(5.4)	13(14.0)	66	3(4.5)	7(10.6)	159(12.4)	8(5.1)	20 (12.6)
*Anser cygnoides*	70	0(0)	5(7.1)	0(0)	0(0)	0(0)	70(5.5)	0(0)	5(7.1)
*Streptopelia chinensis*	85	0(0)	2(2.3)	0(0)	0(0)	0(0)	85(6.6)	0(0)	2(2.3)

* The period from March through October each year

^#^ The period from November through next February each year

^&^ Viral RNA positively detected in at least one organ

Ab: SFTSV-specific antibodies detected by the double antigen sandwich ELISA

Viral RNA: SFTSV-RNA detected by real-time RT-PCR

### Detection of SFTSV-RNA in Larvae and Nymphs of Ticks Collected from Vegetation or Wild Mammals

We first analyzed the pools from questing ticks collected from vegetation. In 2013, we detected SFTSV-RNA positive in two pools each of nymphs [minimal infection rate (MIR), 0.2%] and male adults (0.9%) of *H*. *longicornis*, and one pool of nymphs (1.4%) of *H*. *flava*; in 2014, we detected positive viral RNA in two, ten, six, and five pools of larvae (0.4%), nymphs (0.5%), male adults (0.9%), and female adults (0.7%), respectively, of *H*. *longicornis*, and one pool in nymphs (0.5%) of *H*. *flava* (Table 1). Remarkably, we identified viral RNA of SFTSV in two pools of *H*. *longicornis* larvae that were collected in 2014 from the environment directly and had no blood meal apparently, based on their morphology. Presence of SFTSV-RNA in larvae, collected directly from vegetation, indicates a possible vertical transmission of SFTSV in *H*. *longicornis* in nature. Detection rate of SFTSV-RNA in all stages of *H*. *flava* remained lower, positive viral RNA were only detected in nymphs but not in larvae, suggesting no evidence for the virus to be vertically transmitted in *H*. *flava* at this stage.

We next analyzed the pools from ticks collected from small wild animals. In 2013, we detected SFTSV-RNA positive in two pools each of larvae (MIR 0.7%) and nymphs (0.3%) of *H*. *longicornis*, and one pool of nymphs (1.1%) of *H*. *flava*; in 2014, we detected viral RNA positive in two, seven, two, and two pools of larvae (0.9%), nymphs (0.6%), male adults (1.0%), and female adults (0.5%), respectively, of *H*. *longicornis*, and one pool of nymphs (0.8%) of *H*. *flava* (Table 1). Comparing the two batches from different sources, the overall detection rates for each stage of ticks were similar between vegetation and small animals, and SFTSV-RNA positive samples were only detected in *H*. *longicornis* and *H*. *flava*, but not in any other tick species collected (Tables 1 and S2) Although viral RNA was also identified in multiple pools of larvae and nymphs, we cannot determine with certainty whether the virus was from newly incubated eggs or from their host animals after a blood meal was consumed.

### Detection of SFTSV-RNA and Antibodies in Small Wild Mammals or Migratory Birds

We sought to identify wild amplifying hosts that could harbor the virus after exposure to tick bites, and sampled blood from a variety of captured small wild mammals and birds. During two tick-feeding seasons (March through October in 2013 and 2014), 823 small wild animals, representing eight species of small mammals and two species of wild birds, were captured. Animal blood and tissues were prepared for viral RNA extraction with nested PCR as described earlier, and sera were examined for viral antibodies by a specific sandwich ELISA kit. As shown in Table 2, SFTSV-RNA was detected during the tick-feeding season in *Erinaceus europaeus* (5/93, 5.4%), *Sorex araneus* (2/102, 2.0%), *R*. *fulvescens* (1/46, 2.2%), and *Mus musculus* (1/168, 0.6%); viral antibodies were detected in all species sampled with the highest prevalence in *Erinaceus europaeus* (13/93, 14.0%). Viral antibodies were also detected in *A*. *cygnoides* (5/70, 7.1%) and *S*. *chinensis* (2/85, 2.3%), two migratory birds captured during the tick-feeding season (Table 2). However, we have not detected viral RNA or isolated live virus from the wild birds or their tissues.

We also detected viral RNA and antibodies from blood or tissue samples from animals captured during the off-tick-feeding season from November through the following February. A total of 460 small wild animals including mammals and birds were captured in 2013 and 2014 (November through the following February). Our data indicated that viral RNA of SFTSV was only detected in *Erinaceus europaeus* (3/66, 4.5%) and *Sorex araneus* (1/82, 1.2%) with at least one organ positive, while viral antibodies were detected in *Mus musculus* (1/102, 1.0%), *Rattus norvegicus* (1/66, 1.5%), *Sorex araneus* (2/82, 2.4%), and *Erinaceus europaeus* (7/66, 10.6%). We noted that there were fewer species of animals detected positive for either viral RNA or antibodies in animals captured during the period off the tick-feeding season compared to those during the tick-feeding season. No wild birds tested positive for viral RNA (Table 2).

Using the nested PCR, we further identified SFTSV-RNA in individual tissues or organs from small wild mammals. As shown in Table 3, viral RNA was detected in mammals including *M*. *musculus*, *R*. *fulvescens*, *S*. *araneus*, *and Erinaceus enropaeus*. Spleens were positive for viral RNA in all animals, while other organs or blood varied in their presence of viral RNA. In addition, we showed the viral antibody titers in these animals, captured either during the tick-feeding or off-tick-feeding season, and also showed the collection of the ticks at their various stages on the skin of the captured animals (Table 3). In general, viral RNA was present in organs of animals more frequently during the tick-feeding season than the off- tick-feeding season, although viral antibodies were present in animals captured in both seasons. It is noted that a live SFTSV was isolated in *E*. *europaeus* (JSM-12), with an antibody titer up to 1:512 and captured during the off-tick-feeding season (Table 3), indicating a possibility for a prolonged or persistent SFTSV infection in small mammals in nature.

**Table 3:** Detection of SFTSV RNA/Antibodies and Collection of Attached Ticks in Wild Animals in 2013–2014.

	Wild Animal Species	ID	Animal Organs/Tissues							Titer of Ab	Attached Ticks							
											*H*. *longicornis*				*H*. *flava*			
			Spleen	Heart	Kidney	Liver	Lung	Brain	Blood		Larva	Nymph	F	M	Larva	Nymph	F	M
*Tick-feed-ing Season	***M*. *musculus***	**JSM-1**	**+**	**+**	**+**	**+**	**+**	**-**	**+**	**1:32**	**+**	**-**	**-**	**-**	**-**	**-**	**-**	**-**
	***R*. *fulvescens***	**JSM-2**	**+**	**-**	**+**	**+**	**+**	**-**	**+**	**1:32**	**-**	**+**	**-**	**-**	**+**	**-**	**-**	**-**
	***S*. *araneus***	**JSM-3**	**+**	**+**	**+**	**+**	**+**	**-**	**+**	**1:64**	**-**	**+**	**-**	**-**	**-**	**-**	**-**	**-**
		**JSM-4**	**+**	**-**	**-**	**+**	**+**	**-**	**+**	**1:128**	**+**	**+**	**-**	**-**	**-**	**+**	**-**	**-**
	***E*. *europaeus***	**JSM-5**	**+**	**+**	**+**	**+**	**+**	**-**	**+**	**1:32**	**-**	**+**	**-**	**+**	**-**	**-**	**-**	**-**
		**JSM-6**^&^	**+**	**+**	**+**	**+**	**+**	**-**	**+**	**1:64**	**-**	**+**	**+**	**-**	**-**	**+**	**-**	**-**
		**JSM-7**	**+**	**-**	**-**	**+**	**-**	**-**	**-**	**1:128**	**+**	**+**	**+**	**-**	**-**	**-**	**-**	**-**
		**JSM-8**	**+**	**-**	**+**	**-**	**-**	**-**	**-**	**1:256**	**-**	**+**	**+**	**+**	**-**	**-**	**-**	**-**
		**JSM-9**	**+**	**-**	**+**	**-**	**-**	**-**	**-**	**1:256**	**+**	**+**	**+**	**-**	**+**	**+**	**-**	**-**
#**Off-tick-feeding Season**	***S*. *araneus***	**JSM-10**	**+**	**-**	**-**	**-**	**-**	**-**	**-**	**1:256**	**-**	**-**	**-**	**-**	**-**	**+**	**-**	**-**
	***E*. *europaeus***	**JSM-11**	**+**	**-**	**+**	**-**	**-**	**-**	**-**	**1:256**	**-**	**-**	**-**	**-**	**-**	**-**	**+**	**-**
		**JSM-12**^&^	**+**	**-**	**-**	**-**	**-**	**-**	**-**	**1:512**	**+**	**-**	**-**	**-**	**-**	**-**	**-**	**-**
		**JSM-13**	**+**	**-**	**+**	**+**	**-**	**-**	**+**	**1:1024**	**-**	**+**	**-**	**-**	**-**	**-**	**-**	**-**

*: The period from March through October each year

^#^: The period from November through next February each year

^&^: The animal species from which a live SFTSV was isolated from the spleen

Ab: SFTSV-specific antibodies detected by the double antigen sandwich ELISA

RNA: SFTSV RNA detected by real-time RT-PCR

F: Female adult ticks; M: Male adult ticks

**+**: Viral RNA positive by nested RT-PCR; Attached ticks were collected from the skin of the animals

**-**: Viral RNA negative by nested RT-PCR; No attached ticks were collected from the skin of the animals

Partial S segments (467 bp) of the SFTSV genome were amplified from SFTSV-RNA positive pools of ticks and small animals, and sequences were analyzed with DNA sequencing, which showed 93.9%-99.5% similarity with those from SFTSV strains from humans, goats, and ticks reported previously in China, Korea, and Japan [1,6,7,18].

### Virus Isolation, Sequencing, and Phylogenetic Analysis

Thirty-six strains of SFTSV in Jiangsu were isolated from 2011 through 2014. Among them, 31 were isolated from acute phase sera of SFTS patients, three were from tick pools of *H*. *longicornis*, including one isolate from questing ticks, and two were from spleens of two *Erinaceus europaeus* (one captured during the tick-feeding season and the other off the tick-feeding season)(Table 3).

**Fig 2:**
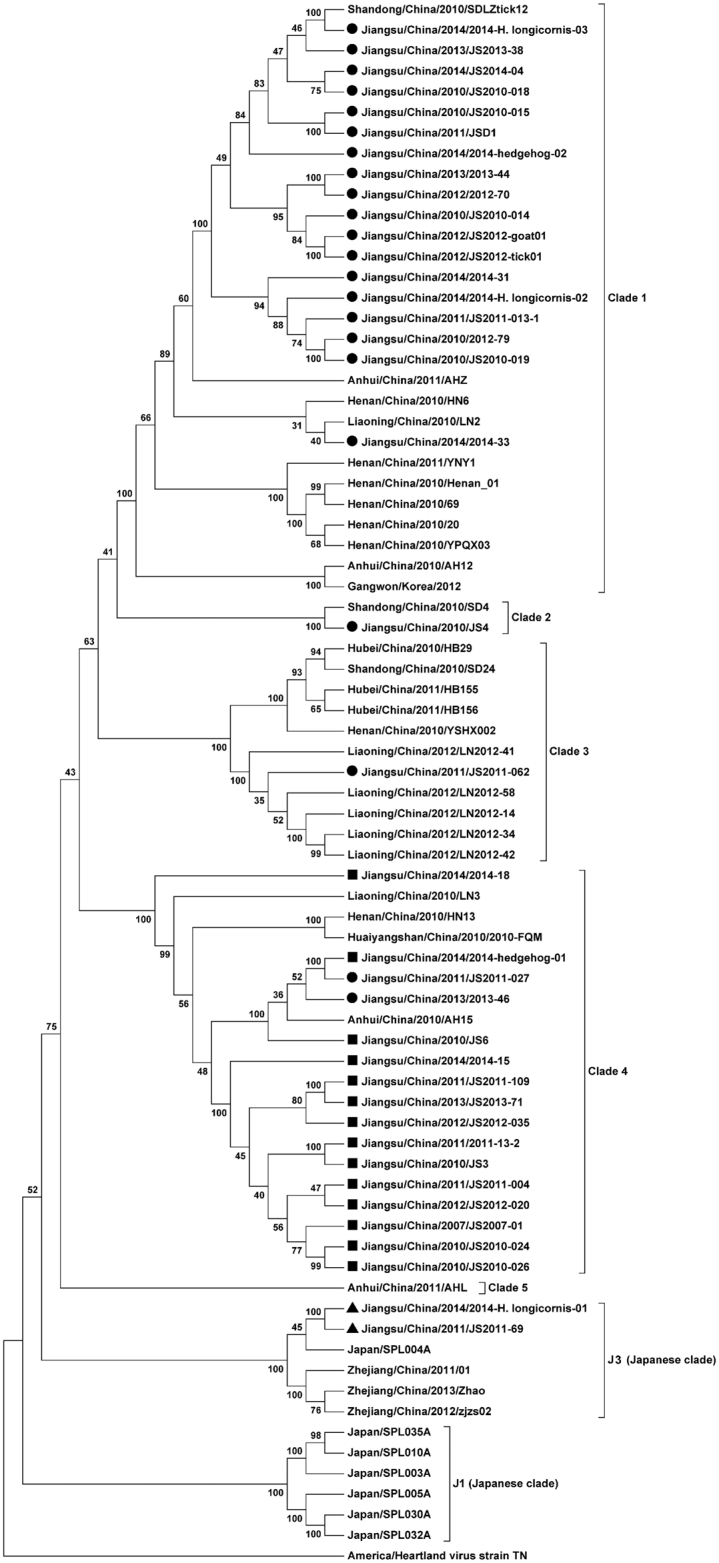
Phylogenetic analysis of SFTSV strains isolated from Jiangsu Province during 2011 and 2014, compared with SFTSV strains from other areas. The phylogenetic tree was constructed by using the Neighbor-Joining method with the MEGA5.1 software. The reliability values indicated at the branch nodes were determined using 1,000 bootstrap replications. Isolated SFTSV strains from places located in the north of the Yangtze River were labeled by black solid circles; Isolated SFTSV strains from places located in the south of the Yangtze river were labeled by black solid squares; Isolated SFTSV strains distributed in Japanese clades were labeled by black solid triangles. Phylogenetic relationship of SFTSV with other bunyaviruses, based on the complete L segment sequences, is shown in the figure.

Whole genome sequencing was performed on 20 SFTSV isolates, including 15 from human serum samples, three from *H*. *longicornis*, and two from *Erinaceus europaeus*. Sequence data have been submitted into the NCBI Genbank (accession numbers KF356517 through KF356552). Using the neighbor joining method in MEGA software, a phylogenetic tree was constructed using the L segment sequences of 42 isolates from Jiangsu province and 35 reference SFTSV strains isolated from other places in China, Korea, and Japan, and showed that all the isolates fall into five genotypes (designated clades 1–5). Two SFTSV strains, one from human (Jiangsu/China/2011/JS2011-69) and the other from ticks (Jiangsu/China/2014/2014-H.longicornis-01) isolated from Yixing County of Jiangsu province, geographically adjacent to Zhejiang province, were clustered, based on the L segment, into the Japanese clade (J3) [25] together with three isolates from a coastal island in Zhejiang province (Fig 2), far away from the sampling sites with survey conducted in this study. The finding suggests that closely related SFTSV strains can be distributed in regions far apart geographically, challenging the theory that the mode of SFTSV transmission is via land movement of infected animals hosting ticks of appropriate species.

## Discussion

The first confirmed SFTS in human was reported in Jiangsu in 2007 [2], and was later determined to be caused by SFTSV, a tick-borne zoonotic virus [1]. Since then, more SFTS cases have been reported in Jiangsu and the neighboring provinces, and SFTSV strains have been isolated from *H*. *longicornis* from vegetation or patients and domestic animals in the villages. Moreover, SFTSV-RNA was detected in *H*. *longicornis* and additional tick species such as *Amblyomma studinarium* and *Ixodes sp*. collected in the endemic region [11,12]. In this study SFTSV RNA was detected in unfed larvae of *H*. *longicornis*, indicating a possibility of a vertical transmission of the virus from infected adult *H*. *longicornis* to their progeny through eggs in nature. Experimentally it was shown recently that viral RNA could be identified in larvae, hatched from eggs laid by infected female adult *H*. *longicornis* [17], indicating that SFTSV can maintain its presence in all stages of this species of ticks and be vertically transmitted, which needs to be further confirmed since no live virus had been successfully isolated from the eggs in this and the above mentioned studies [17].

In this report we collected questing ticks and feeding ticks at 24 sampling sites in the endemic regions of Jiangsu, and examined the presence of SFTSV RNA in captured ticks. We showed that SFTSV RNA was detected in both feeding and questing *H*. *longicornis* and *H*. *flava*. Moreover, one viral strain (2014-H. longicornis-02) was isolated from viral RNA-positive tick pools of questing *H*. *longicornis*, which falls in clade 1 (Fig 2) and is the first to be reported from questing ticks.

SFTSV is thought to circulate in an enzootic tick-vertebrate-tick cycle; however, vertebrate hosts for the virus in nature have yet to be confirmed. Previous surveys have shown that SFTSV infects domestic animals, suggesting that domestic animals can act as amplifying hosts, and might play an important role in viral spread [13,16,23]. However, domestic animals normally have limited lifespans when living near humans, and may not accurately reflect viral ecology and circulation in nature. On the other hand, many species of small wild mammals, such as rodents, ruminants, hedgehogs, weasels, brush-tail possums, and even some bird species, are natural hosts for ticks, whose role in the transmission of arboviruses should not be underestimated [26]. SFTSV RNA and antibodies have been detected in some species of rodents and shrews [27]. In this study, SFTSV RNA was detected in *Erinaceus europaeus* and *Sorex araneus* captured during the tick-feeding and off the tick-feeding seasons. Viral RNA-positive *Erinaceus europaeus* and *Sorex araneus* had a relatively high titer of SFTSV-specific antibodies (Tables 2 and 3). SFTSV-RNA and viral antibodies were detected in more species of animals captured during the tick-feeding season indicating that the virus was transmitted in wild animals via tick biting. However, even though the positive rates with viral antibodies and viral RNA were lower in the off-tick-feeding season animals, the differences appeared not to be striking. Viral antibodies could be maintained in animals for months through the off-tick-feeding season.

Whether or not a prolonged or persistent infection could happen in SFTSV-infected animals remains unknown. In this study two SFTSV strains were isolated from the spleen of two viral RNA-positive *Erinaceus europaeus*(one captured in the tick-feeding season and the other in the off-tick-feeding season). These results suggest that *Erinaceus europaeus* and *Sorex araneus* may act as vertebrate amplifying hosts for the virus. We also noted that viral RNA was detected in the mammals that actually had viral antibodies, suggesting that in these wild animals SFTSV may maintain and replicate in the presence of the specific antibodies. In fact one virus strain was isolated from an animal (*Erinaceus europaeus*) captured during the off-tick-feeding season (JSM-12, Table 3) with a high antibody titer up to 1:512, indicating that the virus might have been replicating in the infected animal for a prolonged period if the animal contracted the virus through tick biting during the tick-feeding season. Prolonged or persistent infection has been found in hantavirus-infected rodents [28,29,30]. Of course merely a virus was isolated from an animal during the off-tick-feeding season may be meaningless for viral transmission. Schmallenberg virus (SBV), also a bunyavirus transmitted by vectors, could be detected in organs of sheep for up to six weeks after an experimental infection. But SBV-RNA could only be found up to ten days in sheep serum after infection, and consequently transmission by *Culicoides*-vectors taking a blood meal at the animal host with a short period of viermia is unlikely [31]. Prolonged or persistent infection of SFTSV in small mammals has to be experimentally studied in order to evaluate the duration of viremia in infected animals. If SFTSV persistent infection with durable viremia is confirmed, these small mammals, such as *Erinaceus europaeus*, would be significant in SFTSV maintenance and transmission in nature.

We sought to further understand viral ecology of SFTSV and its natural circulation mechanism, which is not yet understood. In this study, ticks were collected from both vegetation and captured wild mammals for SFTSV-RNA detection. Remarkably, we identified viral RNA in the earliest developmental stages of *H*. *longicornis* collected from vegetation in 2013 and 2014 (Table 1), including larvae that had not yet fed on a blood meal. These data could be an evidence of the viral transovarial transmission in *H*. *longicornis*. Since SFTSV is present in all life stages of *H*. *longicornis* in nature, and experimentally viral RNA were detected in larvae, nymph, and adult ticks, which had fed on infected mice, and also detected in larvae, hatched from eggs laid from infected female adults [17], we consider *H*. *longicornis* to be a natural reservoir host for SFTSV.

Interestingly, in this study we also detected SFTSV antibodies in two wild bird species, *Anser cygnoides* and *Streptopelia chinensis* (Table 2). We mentioned in our previous study that avian species were susceptible to SFTSV-like virus infection as well [23]. Uukuniemi virus, another phlebovirus which is also an arbovirus transmitted by ticks, can infect and replicate in passerine birds and be carried for long distance transmission [32]. In fact free-living migratory birds of a diverse species play important roles in transmitting arboviruses including equine encephalomyelitis and Sindbis alphaviruses and West Nile and St. Louis encephalitis flaviviruses [33]. Our findings in this study imply that once migratory wild birds are exposed to SFTSV or ticks infected with the virus, the birds could carry the virus, through either their own infection or carrying attached ticks that are infected, to distant regions via flyways. We understand, nevertheless, our results need to be validated by neutralization assays to exclude cross-reactivity. If confirmed, however, we would be able to interpret why a viral strain isolated in Yixing, Jiangsu, could be clustered in the Japanese clade as shown in this study (Fig 2).

Migratory bird transmission of SFTSV may also explain why SFTSV has spread so rapidly in the past few years from the original five provinces in Central China to over 16 provinces, including distant regions in Northeast or Southwest China. A recent report confirmed that *H*. *longicornis* was found on migratory birds whose routes and the distribution of *H*. *longicornis* were concurrent with the occurrence of SFTSV by a phylogenetic analysis [18]. A similar method of transmission might also explain the rapid expansion of the Heartland virus in the United States in the recent years.

In conclusion, we have provided insight into viral ecology of SFTSV, a newly identified phlebovirus in ticks and ticks feeding on small wild mammals and birds in an SFTS endemic area. SFTSV-RNA was identified in larvae of *H*. *longicornis*, indicating that SFTSV could be transmitted transovarially in this tick species, which may serve as a natural reservoir host. Our data did not show, however, that larvae may transmit SFTSV during their blood meal at a mammal or bird in this field study. We also provide evidence to show that small mammals such as *Erinaceus europaeus* and *Sorex araneus*, in which a prolonged infection may occur, can act as amplifying hosts for SFTSV. Antibodies against the virus were detected in migratory birds, suggesting that SFTSV could be transmitted long distances by birds through seasonal flyways. Our findings in this study will help formulate effective measures to deter the further spread of this deadly emerging pathogen.

## Supporting Information

S1 Table Detection of SFTSV RNA in Various Stages of *Haemaphysalis concinna* and *Haemaphysalis doenitzi* Ticks Collected from Vegetation and Wild Animals in 2013–2014 in Jiangsu. *MIR: Minimum infection rate, based on the formula: Number of positive pools/total number of ticks tested. Male: Male adult ticks; Female: Female adult ticks.
(DOCX)

S2 Table Sequences of Isolated SFTSV Strains with Associated Location of Origin, Collection Dates, Host and GenBank Accession Numbers. (DOCX)

## References

[1] Yu XJ, Liang MF, Zhang SY, Liu Y, Li JD, et al (2011) Fever with thrombocytopenia associated with a novel bunyavirus in China. N Engl J Med 364: 1523–1532. 10.1056/NEJMoa1010095 21410387 PMC3113718

[2] Bao CJ, Guo XL, Qi X, Hu JL, Zhou MH, et al (2011) A family cluster of infections by a newly recognized bunyavirus in eastern China, 2007: further evidence of person-to-person transmission. Clin Infect Dis 53: 1208–1214. 10.1093/cid/cir732 22028437

[3] Li Y, Zhou H, Mu D, Yin W, Yu H (2015) [Epidemiological analysis on severe fever with thrombocytopenia syndrome under the national surveillance data from 2011 to 2014, China]. Zhonghua Liu Xing Bing Xue Za Zhi 36: 598–602. 26564632

[4] Zhang L, Sun J, Yan J, Lv H, Chai C, et al (2014) Antibodies against severe fever with thrombocytopenia syndrome virus in healthy persons, China, 2013. Emerg Infect Dis 20: 1355–1357. 10.3201/eid2008.131796 25061813 PMC4111193

[5] Liang S, Bao C, Zhou M, Hu J, Tang F, et al (2014) Seroprevalence and risk factors for severe fever with thrombocytopenia syndrome virus infection in Jiangsu Province, China, 2011. Am J Trop Med Hyg 90: 256–259. 10.4269/ajtmh.13-0423 24343883 PMC3919226

[6] Kim KH, Yi J, Kim G, Choi SJ, Jun KI, et al (2013) Severe fever with thrombocytopenia syndrome, South Korea, 2012. Emerg Infect Dis 19: 1892–1894. 10.3201/eid1911.130792 24206586 PMC3837670

[7] Takahashi T, Maeda K, Suzuki T, Ishido A, Shigeoka T, et al (2014) The first identification and retrospective study of Severe Fever with Thrombocytopenia Syndrome in Japan. J Infect Dis 209: 816–827. 10.1093/infdis/jit603 24231186 PMC7107388

[8] McMullan LK, Folk SM, Kelly AJ, MacNeil A, Goldsmith CS, et al (2012) A new phlebovirus associated with severe febrile illness in Missouri. N Engl J Med 367: 834–841. 10.1056/NEJMoa1203378 22931317

[9] Ding F, Zhang W, Wang L, Hu W, Soares Magalhaes RJ, et al (2013) Epidemiologic features of severe fever with thrombocytopenia syndrome in China, 2011–2012. Clin Infect Dis 56: 1682–1683. 10.1093/cid/cit100 23429379

[10] Liu Q, He B, Huang SY, Wei F, Zhu XQ (2014) Severe fever with thrombocytopenia syndrome, an emerging tick-borne zoonosis. Lancet Infect Dis 14: 763–772. 10.1016/S1473-3099(14)70718-2 24837566

[11] Wang S, Li J, Niu G, Wang X, Ding S, et al (2015) SFTS virus in ticks in an endemic area of China. Am J Trop Med Hyg 92: 684–689. 10.4269/ajtmh.14-0008 25711611 PMC4385759

[12] Yun SM, Lee WG, Ryou J, Yang SC, Park SW, et al (2014) Severe fever with thrombocytopenia syndrome virus in ticks collected from humans, South Korea, 2013. Emerg Infect Dis 20: 1358–1361. 10.3201/eid2008.131857 25061851 PMC4111194

[13] Ding S, Yin H, Xu X, Liu G, Jiang S, et al (2014) A cross-sectional survey of severe fever with thrombocytopenia syndrome virus infection of domestic animals in Laizhou City, Shandong Province, China. Jpn J Infect Dis 67: 1–4. 24451093 10.7883/yoken.67.1

[14] Park SW, Song BG, Shin EH, Yun SM, Han MG, et al (2014) Prevalence of severe fever with thrombocytopenia syndrome virus in Haemaphysalis longicornis ticks in South Korea. Ticks Tick Borne Dis 5: 975–977. 10.1016/j.ttbdis.2014.07.020 25164614

[15] Savage HM, Godsey MS Jr., Lambert A, Panella NA, Burkhalter KL, et al (2013) First detection of heartland virus (Bunyaviridae: Phlebovirus) from field collected arthropods. Am J Trop Med Hyg 89: 445–452. 10.4269/ajtmh.13-0209 23878186 PMC3771279

[16] Niu G, Li J, Liang M, Jiang X, Jiang M, et al (2013) Severe fever with thrombocytopenia syndrome virus among domesticated animals, China. Emerg Infect Dis 19: 756–763. 10.3201/eid1905.120245 23648209 PMC3647489

[17] Luo LM, Zhao L, Wen HL, Zhang ZT, Liu JW, et al (2015) Haemaphysalis longicornis Ticks as Reservoir and Vector of Severe Fever with Thrombocytopenia Syndrome Virus in China. Emerg Infect Dis 21: 1770–1776. 10.3201/eid2110.150126 26402039 PMC4593435

[18] Yun Y, Heo ST, Kim G, Hewson R, Kim H, et al (2015) Phylogenetic Analysis of Severe Fever with Thrombocytopenia Syndrome Virus in South Korea and Migratory Bird Routes Between China, South Korea, and Japan. Am J Trop Med Hyg. 10.4269/ajtmh.15-0047 PMC4559681 26033016

[19] Li Z, Cui L, Zhou M, Qi X, Bao C, et al (2013) Development and application of a one-step real-time RT-PCR using a minor-groove-binding probe for the detection of a novel bunyavirus in clinical specimens. J Med Virol 85: 370–377. 10.1002/jmv.23415 23212930

[20] Mills J C J, Ksiazek T, Peters C, Velleca W, (1995) Methods for Trapping and Sampling Small Mammals for Virologic Testing. Atlanta, GA: United States Department of Health and Human Services.

[21] Torres-Perez F, Navarrete-Droguett J, Aldunate R, Yates TL, Mertz GJ, et al (2004) Peridomestic small mammals associated with confirmed cases of human hantavirus disease in southcentral Chile. Am J Trop Med Hyg 70: 305–309. 15031522

[22] Jiao Y, Zeng X, Guo X, Qi X, Zhang X, et al (2012) Preparation and evaluation of recombinant severe fever with thrombocytopenia syndrome virus nucleocapsid protein for detection of total antibodies in human and animal sera by double-antigen sandwich enzyme-linked immunosorbent assay. J Clin Microbiol 50: 372–377. 10.1128/JCM.01319-11 22135253 PMC3264160

[23] Xing Z, Schefers J, Schwabenlander M, Jiao Y, Liang M, et al (2013) Novel bunyavirus in domestic and captive farmed animals, Minnesota, USA. Emerg Infect Dis 19: 1487–1489. 10.3201/eid1909.130165 23966016 PMC5485073

[24] Huang XY, Du YH, Li XL, Ma H, Man RQ, et al (2012) [Establishment of indirect immunofluorescence assay (IFA) for detection of IgG antibody against new bunyavirus]. Zhonghua Yu Fang Yi Xue Za Zhi 46: 165–168. 22490201

[25] Yoshikawa T, Shimojima M, Fukushi S, Tani H, Fukuma A, et al (2015) Phylogenetic and Geographic Relationships of Severe Fever With Thrombocytopenia Syndrome Virus in China, South Korea, and Japan. J Infect Dis 212: 889–898. 10.1093/infdis/jiv144 25762790

[26] Ostfeld RS, Levi T, Jolles AE, Martin LB, Hosseini PR, et al (2014) Life history and demographic drivers of reservoir competence for three tick-borne zoonotic pathogens. PLoS One 9: e107387 10.1371/journal.pone.0107387 25232722 PMC4169396

[27] Liu JW, Wen HL, Fang LZ, Zhang ZT, He ST, et al (2014) Prevalence of SFTSV among Asian house shrews and rodents, China, January-August 2013. Emerg Infect Dis 20: 2126–2128. 10.3201/eid2012.141013 25418111 PMC4257798

[28] Mills JN, Schmidt K, Ellis BA, Calderon G, Enria DA, et al (2007) A longitudinal study of hantavirus infection in three sympatric reservoir species in agroecosystems on the Argentine Pampa. Vector Borne Zoonotic Dis 7: 229–240. 17627443 10.1089/vbz.2006.0614

[29] Meyer BJ, Schmaljohn CS (2000) Persistent hantavirus infections: characteristics and mechanisms. Trends Microbiol 8: 61–67. 10664598 10.1016/s0966-842x(99)01658-3

[30] Easterbrook JD, Klein SL (2008) Immunological mechanisms mediating hantavirus persistence in rodent reservoirs. PLoS Pathog 4: e1000172 10.1371/journal.ppat.1000172 19043585 PMC2584234

[31] Wernike K, Hoffmann B, Breard E, Botner A, Ponsart C, et al (2013) Schmallenberg virus experimental infection of sheep. Vet Microbiol 166: 461–466. 10.1016/j.vetmic.2013.06.030 23972950

[32] Saikku P (1974) Passerine birds in the ecology of Uukuniemi virus. Med Biol 52: 98–103. 4837432

[33] Hubalek Z (2004) An annotated checklist of pathogenic microorganisms associated with migratory birds. J Wildl Dis 40: 639–659. 15650082 10.7589/0090-3558-40.4.639

