# Supplementary material for: Ecology of the Tick-Borne Phlebovirus Causing Severe Fever with Thrombocytopenia Syndrome in an Endemic Area of China
Source: PLoS Negl Trop Dis. 2016 Apr 1;10(4):e0004574. doi: 10.1371/journal.pntd.0004574 (PMC4818090; doi:10.1371/journal.pntd.0004574)
Supplement: S1 Table — *MIR: Minimum infection rate, based on the formula: Number of positive pools/total number of ticks tested. Male: Male adult ticks; Female: Female adult ticks. (DOCX) [file pntd.0004574.s001.docx]

Table S1. Sequences of Isolated SFTSV Strains with Associated Location, Collection Dates,
Host and GenBank Accession Numbers

| SFTSV strain | Time of isolation | Host | Geographic origin | GenBank accession NO. | | |
| --- | --- | --- | --- | --- | --- | --- |
|  |  |  |  | L segment | M segment | S segment |
| JS2007-01 | 2007 | Human | Jiangsu | JF837593 | JF837594 | JF837595 |
| JS3-2010 | 2010 | Human | Jiangsu | HQ141601 | HQ141602 | HQ141603 |
| JS4-2010 | 2010 | Human | Jiangsu | HQ141604 | HQ141605 | HQ141606 |
| JS6-2010 | 2010 | Human | Jiangsu | HQ830169 | HQ830170 | HQ830171 |
| JS24-2010 | 2010 | Human | Jiangsu | HQ830163 | HM802201 | HQ830165 |
| JS26-2010 | 2010 | Human | Jiangsu | HQ830166 | HQ830167 | HQ830168 |
| JS2010-014 | 2010 | Human | Jiangsu | JQ317169 | JQ317170 | JQ317171 |
| JS2010-015 | 2010 | Human | Jiangsu | JQ317172 | JQ317173 | JQ317174 |
| JS2010-018 | 2010 | Human | Jiangsu | JQ317175 | JQ317176 | JQ317177 |
| JS2010-019 | 2010 | Human | Jiangsu | JQ317178 | JQ317179 | JQ317180 |
| JSD1-2011 | 2011 | Dog | Jiangsu | JF267783 | JF267784 | JF267784 |
| JS2011-004 | 2011 | Human | Jiangsu | KC505123 | KC505124 | KC505125 |
| JS2011-013-1 | 2011 | Human | Jiangsu | KC505126 | KC505127 | KC505128 |
| JS2011-027 | 2011 | Human | Jiangsu | KC505129 | KC505130 | KC505131 |
| JS2011-062 | 2011 | Human | Jiangsu | KC505135 | KC505136 | KC505137 |
| JS2011-109 | 2011 | Human | Jiangsu | KC505138 | KC505139 | KC505140 |
| JS2012-020 | 2012 | Human | Jiangsu | KC505141 | KC505142 | KC505143 |
| JS2012-035 | 2012 | Human | Jiangsu | KC505144 | KC505145 | KC505146 |
| JS2012-goat01 | 2012 | Goat | Jiangsu | KC473537 | KC473538 | KC473539 |
| JS2012-tick01 | 2012 | Haemaphysalis | Jiangsu | KC473540 | KC473541 | KC473542 |
| JS2011-13-2 | 2011 | Human | Jiangsu | KR230750 | KR230770 | KR230790 |
| JS2011-69 | 2011 | Human | Jiangsu | KR230751 | KR230771 | KR230791 |
| JS2012-79 | 2012 | Human | Jiangsu | KR230752 | KR230772 | KR230792 |
| JS2013-44 | 2013 | Human | Jiangsu | KR230753 | KR230773 | KR230793 |
| JS2013-46 | 2013 | Human | Jiangsu | KR230754 | KR230774 | KR230794 |
| JS2013-52(38) | 2013 | Human | Jiangsu | KR230755 | KR230775 | KR230795 |
| JS2013-69 | 2013 | Human | Jiangsu | KR230756 | KR230776 | KR230796 |
| JS2013-71 | 2013 | Human | Jiangsu | KR230757 | KR230777 | KR230797 |
| JS2014-03(04) | 2014 | Human | Jiangsu | KR230758 | KR230778 | KR230798 |
| JS2014-15 | 2014 | Human | Jiangsu | KR230759 | KR230779 | KR230799 |
| JS2014-16 | 2014 | Human | Jiangsu | KR230760 | KR230780 | KR230800 |
| JS2014-18 | 2014 | Human | Jiangsu | KR230761 | KR230781 | KR230801 |
| JS2014-31 | 2014 | Human | Jiangsu | KR230762 | KR230782 | KR230802 |
| JS2014-33 | 2014 | Human | Jiangsu | KR230763 | KR230783 | KR230803 |
| JS2014-39 | 2014 | Human | Jiangsu | KR230764 | KR230784 | KR230804 |
| 2014-H.longicornis-01 | 2014 | *H.longicornis* | Jiangsu | KR230765 | KR230785 | KR230805 |
| 2014-H.longicornis-02 | 2014 | *H.longicornis* | Jiangsu | KR230766 | KR230786 | KR230806 |
| 2014-H.longicornis-03 | 2014 | *H.longicornis* | Jiangsu | KR230767 | KR230787 | KR230807 |
| JS2014-hedgehog-01 | 2014 | *Erinaceus europaeus Linnaeus* | Jiangsu | KR230768 | KR230788 | KR230808 |
| JS2014-hedgehog-02 | 2014 | *Erinaceus europaeus Linnaeus* | Jiangsu | KR230769 | KR230789 | KR230809 |
| AH12 | 2010 | Human | Anhui | HQ116417 | HQ141590 | HQ141591 |
| AH15 | 2010 | Human | Anhui | HQ141592 | HQ141593 | HQ141594 |
| AHZ | 2011 | Human | Anhui | JQ670929 | JQ670931 | JQ670933 |
| Gangwon | 2012 | Human | Korea | KF358691 | KF358692 | KF358693 |
| HB155 | 2011 | Human | Hubei | JQ733564 | JQ733563 | JQ733565 |
| HB156 | 2011 | Human | Hubei | JQ733567 | JQ733566 | JQ733568 |
| HB29 | 2010 | Human | Hubei | KP202163 | KP202164 | KP202165 |
| CHN-01 | 2010 | Human | Henan | HQ642766 | HQ642767 | HQ642768 |
| CHN-20 | 2010 | Human | Henan | JF682773 | JF682774 | JF682775 |
| CHN-69 | 2010 | Human | Henan | JF682776 | JF682777 | JF682778 |
| HN13 | 2010 | Human | Henan | HQ141598 | HQ141599 | HQ141600 |
| HN6 | 2010 | Human | Henan | HQ141595 | HQ141596 | HQ141597 |
| YNY1 | 2011 | Human | Henan | KF356552 | KF356538 | KF356526 |
| YPQX03 | 2010 | Human | Henan | KF356550 | KF356539 | KF356527 |
| YSHX002 | 2010 | Human | Henan | KF356551 | KF356540 | KF356528 |
| FQM | 2010 | Human | Huaiyangshan | HQ419227 | HQ419236 | HQ419240 |
| SPL003A | unknown | Human | Japan | AB817980 | AB817988 | AB817996 |
| SPL004A | unknown | Human | Japan | AB817981 | AB817989 | AB817997 |
| SPL005A | unknown | Human | Japan | AB817982 | AB817990 | AB817998 |
| SPL010A | unknown | Human | Japan | AB817983 | AB817991 | AB817999 |
| SPL030A | unknown | Human | Japan | AB817984 | AB817992 | AB818000 |
| SPL032A | unknown | Human | Japan | AB817985 | AB817993 | AB818001 |
| SPL035A | unknown | Human | Japan | AB817986 | AB817994 | AB818002 |
| LN2 | 2010 | Human | Liaoning | HQ141607 | HQ141608 | HQ141609 |
| LN3 | 2010 | Human | Liaoning | HQ141610 | HQ141611 | HQ141612 |
| LN2012-14 | 2012 | Human | Liaoning | KF887441 | KF887436 | KF887431 |
| LN2012-34 | 2012 | Human | Liaoning | KF887442 | KF887437 | KF887432 |
| LN2012-41 | 2012 | Human | Liaoning | KF887443 | KF887438 | KF887433 |
| LN2012-42 | 2012 | Human | Liaoning | KF887444 | KF887439 | KF887434 |
| LN2012-58 | 2012 | Human | Liaoning | KF887445 | KF887440 | KF887435 |
| SD24 | 2010 | Human | Shandong | HM802200 | HM802201 | HM802205 |
| SD4 | 2010 | Human | Shandong | HM802202 | HM802203 | HM802204 |
| zjzs02 | 2012 | Human | Zhejiang | KC189855 | KC189856 | KC189857 |
| SDLZtick12 | 2010 | Tick | Shandong | JQ684871 | JQ684872 | JQ684873 |
| Zhejiang-01 | 2011 | Human | Zhejiang | KJ597825 | KJ597824 | KJ597823 |
| Zhao | 2013 | Human | Zhejiang | KF374682 | KF374683 | KF374684 |
