# Supplementary material for: Ecology of the Tick-Borne Phlebovirus Causing Severe Fever with Thrombocytopenia Syndrome in an Endemic Area of China
Source: PLoS Negl Trop Dis. 2016 Apr 1;10(4):e0004574. doi: 10.1371/journal.pntd.0004574 (PMC4818090; doi:10.1371/journal.pntd.0004574)
Supplement: S2 Table — (DOCX) [file pntd.0004574.s002.docx]

**Table S2. Detection of SFTSV RNA in Various Stages of *Haemaphysalis* *concinna* and *Haemaphysalis* *doenitzi* Ticks Collected from Vegetation and Wild Animals in 2013-2014 in Jiangsu**

| **Sources** | **Species** | | **2013** | | | |  | **2014** | | | |
| --- | --- | --- | --- | --- | --- | --- | --- | --- | --- | --- | --- |
|  |  |  | **Ticks** | **Pools** | **Positive Pools** | **MIR (%)*** |  | **Ticks** | **Pools** | **Positive Pools** | **MIR (%)** |
| **Vegetation** | ***Haemaphysalis***  ***concinna*** | **Larva** | 0 | 0 | 0 | 0 |  | 16 | 2 | 0 | 0 |
|  |  | **Nymph** | 50 | 4 | 0 | 0 |  | 48 | 4 | 0 | 0 |
|  |  | **Male** | 46 | 6 | 0 | 0 |  | 6 | 2 | 0 | 0 |
|  |  | **Female** | 24 | 4 | 0 | 0 |  | 10 | 2 | 0 | 0 |
|  |  | **Subtotal** | 120 | 14 | 0 | 0 |  | 80 | 10 | 0 | 0 |
|  | ***Haemaphysalis doenitzi*** | **Larva** | 6 | 2 | 0 | 0 |  | 0 | 0 | 0 | 0 |
|  |  | **Nymph** | 32 | 4 | 0 | 0 |  | 8 | 2 | 0 | 0 |
|  |  | **Male** | 12 | 2 | 0 | 0 |  | 0 | 0 | 0 | 0 |
|  |  | **Female** | 0 | 0 | 0 | 0 |  | 0 | 0 | 0 | 0 |
|  |  | **Subtotal** | 50 | 8 | 0 | 0 |  | 8 | 2 | 0 | 0 |
| **Animals** | ***Haemaphysalis***  ***concinna*** | **Larva** | 14 | 2 | 0 | 0 |  | 10 | 2 | 0 | 0 |
|  |  | **Nymph** | 24 | 4 | 0 | 0 |  | 68 | 6 | 0 | 0 |
|  |  | **Male** | 0 | 0 | 0 | 0 |  | 24 | 4 | 0 | 0 |
|  |  | **Female** | 0 | 0 | 0 | 0 |  | 26 | 4 | 0 | 0 |
|  |  | **Subtotal** | 38 | 6 | 0 | 0 |  | 128 | 22 | 0 | 0 |
|  | ***Haemaphysalis doenitzi*** | **Larva** | 26 | 1 | 0 | 0 |  | 0 | 0 | 0 | 0 |
|  |  | **Nymph** | 28 | 2 | 0 | 0 |  | 12 | 2 | 0 | 0 |
|  |  | **Male** | 0 | 0 | 0 | 0 |  | 6 | 2 | 0 | 0 |
|  |  | **Female** | 0 | 0 | 0 | 0 |  | 10 | 2 | 0 | 0 |
|  |  | **Subtotal** | 54 | 3 | 0 | 0 |  | 28 | 6 | 0 | 0 |
| **Total** |  | **Larva** | 46 | 5 | 0 | 0 |  | 26 | 4 | 0 | 0 |
|  |  | **Nymph** | 134 | 14 | 0 | 0 |  | 136 | 14 | 0 | 0 |
|  |  | **Male** | 58 | 8 | 0 | 0 |  | 36 | 8 | 0 | 0 |
|  |  | **Female** | 24 | 4 | 0 | 0 |  | 46 | 8 | 0 | 0 |
|  |  | **Total** | 262 | 31 | 0 | 0 |  | 244 | 34 | 0 | 0 |

*MIR: Minimum infection rate, based on the formula: Number of positive pools/total number of ticks tested.

Male: Male adult ticks; Female: Female adult ticks
